# Maslinic acid potentiates the anti-tumor activity of tumor necrosis factor α by inhibiting NF-κB signaling pathway

**DOI:** 10.1186/1476-4598-9-73

**Published:** 2010-04-06

**Authors:** Chenghai Li, Zhengfeng Yang, Chunyan Zhai, Wenwei Qiu, Dali Li, Zhengfang Yi, Lei Wang, Jie Tang, Min Qian, Jian Luo, Mingyao Liu

**Affiliations:** 1 The Institute of Biomedical Sciences and School of Life Sciences, East China Normal University, Shanghai 200241, China; 2 Institute of Medicinal Chemistry, Department of Chemistry, East China Normal University, Shanghai 200241, China; 3 Institute of Biosciences and Technology, Texas A&M Health Science Center, Houston, Texas 77030, USA

## Abstract

**Background:**

Tumor necrosis factor alpha (TNFα) has been used to treat certain tumors in clinic trials. However, the curative effect of TNFα has been undermined by the induced-NF-κB activation in many types of tumor. Maslinic acid (MA), a pharmacological safe natural product, has been known for its important effects as anti-oxidant, anti-inflammatory, and anti-viral activities. The aim of this study was to determine whether MA potentiates the anti-tumor activity of TNFα though the regulation of NF-κB activation.

**Results:**

In this study, we demonstrate that MA significantly enhanced TNFα-induced inhibition of pancreatic cancer cell proliferation, invasion, and potentiated TNFα-induced cell apoptosis by suppressing TNFα-induced NF-κB activation in a dose- and time-dependent manner. Addition of MA inhibited TNFα-induced IκBα degradation, p65 phosphorylation, and nuclear translocation. Furthermore, MA decreased the expression levels of NF-κB-regulated genes, including genes involved in tumor cell proliferation (Cyclin D1, COX-2 and c-Myc), apoptosis (Survivin, Bcl-2, Bcl-xl, XIAP, IAP-1), invasion (MMP-9 and ICAM-1), and angiogenesis (VEGF). In athymic nu/nu mouse model, we further demonstrated that MA significantly suppressed pancreatic tumor growth, induced tumor apoptosis, and inhibited NF-κB-regulated anti-apoptotic gene expression, such as Survivin and Bcl-xl.

**Conclusions:**

Our data demonstrate that MA can potentiate the anti-tumor activities of TNFα and inhibit pancreatic tumor growth and invasion by activating caspase-dependent apoptotic pathway and by suppressing NF-κB activation and its downstream gene expression. Therefore, MA together with TNFα could be new promising agents in the treatment of pancreatic cancer.

## Background

Pancreatic cancer is an exceptionally devastating and incurable disease, which is becoming the fourth killer of patients with cancer [1] It was estimated that 34,300 people died of pancreatic cancer in the United States and 40,000 in Europe in 2008 alone [1,2]. The treatment of pancreatic cancer has largely been unsuccessful due to its uncontrolled growth, high rate metastasis, and common resistance to conventional approaches including surgery, radiation and/or chemotherapy [3-5]. Therefore, there is a need for development of new and effective chemotherapeutic agents, which can target multiple signaling pathways to induce responsiveness of pancreatic cancer cells to death signals [6,7].

TNFα, a key proinflammatory factor, has been shown to have anti-tumor activity in several preclinical models and in non-comparative clinical trials [8-12]. However, the curative effect seems not perfect as anticipated due to its systemic cytotoxicities and resistance to tumor cells, which prevents TNFα from becoming an effective anticancer agent [10,13]. Recent studies have shown that the abnormal activation of NF-κB is the main reason for TNFα tolerance in many types of tumor [8,10,13,14]. When binding to its receptor (tumor necrosis factor receptor, TNFR) on the cells, TNFα can induce apoptosis via recruiting TNFR-associated death domain protein (TRADD), FADD, and Caspase-8, and then modulate Caspase-9 and Caspase-3. The activated Caspase-3 can induce poly (ADP-ribose) polymerase (PARP) cleavage and switch on cell apoptosis programs. On the other hand, bound receptor TNFR has been shown to activate IκBα kinase (IKK). IKK, in turn, phosphorylates the IκBα protein, resulting in the ubiquitination and dissociation of IκBα from NF-κB complex, and eventually leading to the degradation of IκBα by the proteosome. The activated NF-κB is then translocated into the nucleus where it binds to specific sequences of DNA and activates the expression of some anti-apoptosis genes, such as Bcl-2, Survivin, XIAP, and IAPs [15,16]. Therefore, agents that can suppress TNFα-induced NF-κB activation, and at the same time enhance TNFα-induced cell apoptotic activation will significantly improve the anti-tumor activities of TNFα [8,10,13].

Natural products have been a successful source of therapeutic agents and drug leads. MA, a pentacyclic triterpene acid, is widely present in dietary plants, especially abundant in olive fruit skins. The compound has attracted much interest due to its proven pharmacological safety and its many biological activities such as anti-inflammation[17], anti-virus [18], anti-oxidation [17,19], and anti-diabetogenic activities [20,21]. Recently, some studies have shown that MA has moderate anti-cancer activities in colon cancer [22] and astrocytoma cell [23]. However, the mechanisms of MA action in inflammation and cancers are still not clear, and the synergistic effects of MA and TNFα in inhibiting tumor growth and proliferation have not been investigated.

In this study, we demonstrated that MA greatly potentiated the inhibitory effect of TNFα on the growth of pancreatic cancer through activation of apoptosis. MA activates TNFα-induced caspase and PARP apoptotic signaling pathway while suppresses TNFα-induced NF-κB activation in a dose-dependent manner. Moreover, MA inhibited TNFα-induced IκBα degradation, p65 phosphorylation and nuclear translocation, as well as NF-κB mediated gene expression. Finally, *in vivo *athymic nu/nu mouse model, MA suppressed pancreatic tumor growth and induced tumor cell apoptosis by inhibiting NF-κB-regulated anti-apoptosis genes, such as Survivin and Bcl-xl. Therefore, our results demonstrated that MA potentiated the efficacy of TNFα susceptibility to pancreatic tumor by enhancing caspase apoptotic signaling pathway and by inhibiting NF-κB activation and its down-stream gene expression.

## Results

### MA enhanced TNFa-mediated inhibition of pancreatic cancer cell proliferation

**Figure 1:**
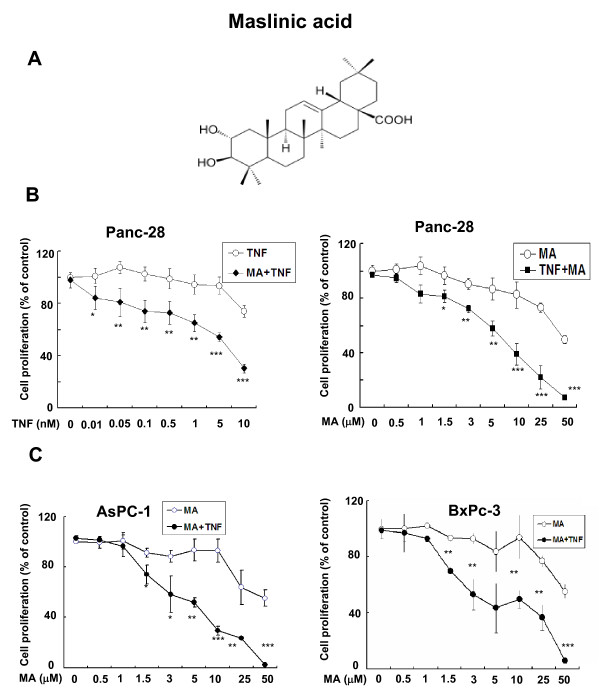
**MA enhances cell proliferation inhibition mediated by TNFα**. (**A**) Structure of Maslinic Acid (MA) [(2α, 3β)-2, 3-dihydroxyolean-12-en-28-oic acid]. (**B**) MA potentiates TNFα-mediated cell growth inhibitory in different pancreatic cancer cell lines. TNFα inhibits cell proliferation in a dose-dependent manner and this inhibitory effect was significantly increased by 10 microM MA (left). And MA inhibits cell proliferation in a dose-dependent manner in the presence of a non-growth inhibitory dose of TNFα (0.1 nM) (right). Cell proliferation was evaluated by MTS method as suggested by the manufacture. Points, means of three experiments carried out in triplicate; *bars*, SD. * *P *< 0.05,**, *P *< 0.01, ***, *P *< 0.001 versus MA or TNF alone treated group. (**C**) MA potentiates TNFα-mediated cell growth inhibition in AsPC-1 and BxPC-3 pancreatic cancer cell lines in a dose-dependent manner. Cell growth was evaluated by MTS method. Points, means of three experiments carried out in triplicate; *bars*, SD. * *P *< 0.05, **, *P *< 0.01, ***, *P *< 0.001 versus MA or TNF.alone treated group.

To determine the effects of MA (Fig. 1A), a recently discovered triterpenoid isolated as the main compound from olive-skin pomace on pancreatic cancer cell growth, we treated Panc-28 pancreatic cancer cells with different concentration of TNFα, MA or together and then measured cell proliferation by MTS assay. Our data indicated that TNFα or MA alone only had moderate inhibitory effect on the growth of Panc-28 cells (Fig. 1B, White dot). However, in the presence of a non-growth inhibitory dose of TNFα (0.1 nM) or MA (10 microM), MA together with TNFα was highly effective on Panc-28 cells proliferation in a dose-dependent manner (Fig. 1B, black dot). Similar effects were also observed in 2 other pancreatic cancer cell lines, AsPC-1 and BxPC-3 (Fig. 1C), suggesting that MA is an effective agent in suppressing pancreatic cancer cell proliferation in the presence of TNFα. These results showed that MA and TNFα together could effectively suppress pancreatic cancer cell growth.

### MA potentiates pancreatic cancer cell apoptosis induced by TNFα

**Figure 2:**
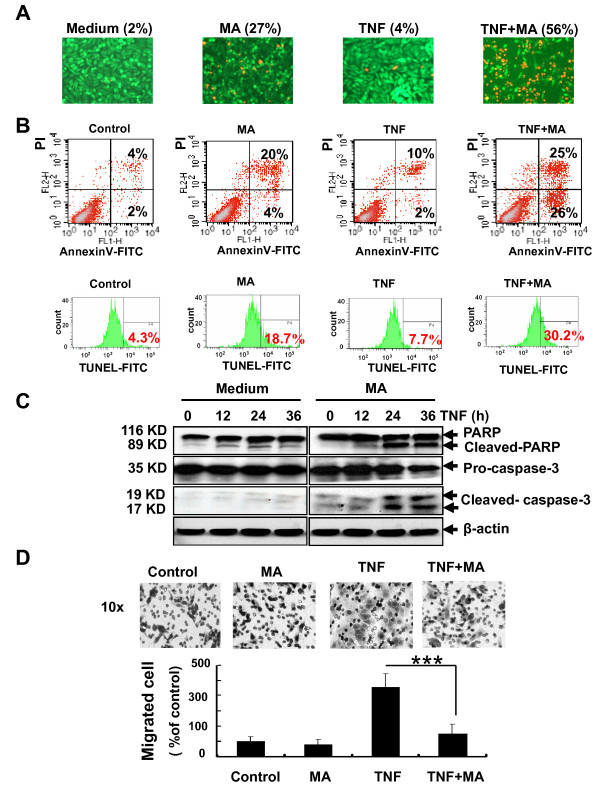
**MA potentiates TNFα-induced pancreatic cancer cell apoptosis and suppresses TNFα-induced invasion**. (**A**) MA inhibits TNFα-induced cell viability by Live/Dead assay. Panc-28 cells were pretreated with or without 25 microM MA for 12 h, and then incubated with 0.1 nM TNFα for 24 h. The live (green) or dead (red) cells were stained with Live and dead assay kit as mentioned in Materials and Methods. Magnification, ×10. (**B**) MA potentiates cell apoptosis induced by TNFα using annexin V staining (top) and TUNEL staining (bottom). Panc-28 cells were pretreated with or without 25 microM MA for 6 (or 12) h, and incubated with or without 0.1 nM TNFα for another 12 (or 24) h, then stained with annexin V-FITC (or TUNEL) and analyzed by flow cytometry as mentioned in Materials and Methods. (**C**) MA potentiates the cleavage of PARP and caspase-3. Panc-28 cells were pretreated with or without 25 microM MA for 6 h, and then incubated with or without 0.1 nM TNFα for the indicated time. Cell lysates were harvested and analyzed by Western blot with the indicated antibodies. (**D**) MA suppresses pancreatic cancer cell invasion induced by TNFα. Panc-28 cells were seeded onto the top chambers of the transwell. After treated with 10 microM MA for 12 h, the number of cells invaded the transwell was counted as described in Materials and Methods. *Columns*, mean; *bars*, SD. Magnification, ×10.

To understand how MA suppressed pancreatic cancer cell proliferation, we examined whether MA affected the viability of pancreatic cancer cells by Live/Dead assays, which determines intracellular esterase activity and membrane integrity to identify live or dead cells. As shown in Fig. 2A, TNFα alone induced cell death from 2 ± 1.02% to 4 ± 1.31% (red signal), and MA alone induced cell death from 2 ± 1.02% to 27 ± 3.58%. When used together, MA and TNFα synergistically induced cell death to 56 ± 5.34% in Panc-28 cells, indicating that MA can significantly decrease pancreatic cancer cell viability induced by TNFα *in vitro*.

To further examine whether the cytotoxicity of MA was caused through cell apoptosis, we analyzed the apoptotic effect of MA using Annexin V staining and TUNEL assay. The Annexin V staining determines the early apoptosis events, in which the membrane phospholipid phosphatidylserine moves from cell cytoplasmic interface to the extracellular surface [16]. Our data indicated that MA together with TNFα potentiated cell apoptosis from 6.0 ± 2.01% to 51 ± 5.32% in pancreatic cancer cells (Fig. 2B, top). Similar results were obtained by TUNEL assay, in which MA enhanced TNFα-induced apoptosis from 7.7 ± 0.89% to 32.2 ± 0.98%, suggesting a synergistic effect of MA with TNFα in inducing cell apoptosis (Fig. 2B, bottom). Therefore, our results demonstrated that combination of MA with TNFα could significantly affect pancreatic cancer cell viability and induce cancer cell apoptosis.

### MA potentiates the TNFα-induced activation of caspase-3 and PARP in pancreatic cancer cells

To confirm the synergistic effect of MA and TNFα on cell apoptosis, we examined wheather MA affects TNFα-induced activation of caspase apoptotic pathway in pancreatic cancer cells by measuring the marker proteins, PARP and caspase-3, using Western blot analysis. As shown in Fig. 2C, the accumulation of cleavaged PARP and caspase-3 was markedly increased during incubation with TNFα and MA together, indicating that MA can facilitate the activation of caspase and PARP apoptotic pathway induced by TNFα.

### MA suppresses TNFα-induced pancreatic cancer cell invasion

The lethal nature of pancreatic cancer stems from its high invasion ability and propensity to rapidly metastasis to the lymphatic system and distant organs [3-5]. To determine the effects of MA on pancreatic cancer cell invasion, we performed the transwell invasion assay and found that MA can markedly inhibit TNFα-induced Panc-28 pancreatic cancer cell invasion, while MA alone had no significantly inhibitory activity (Fig. 2D).

### MA inhibits TNFα-induced NF-κB activity in pancreatic cancer cells

**Figure 3:**
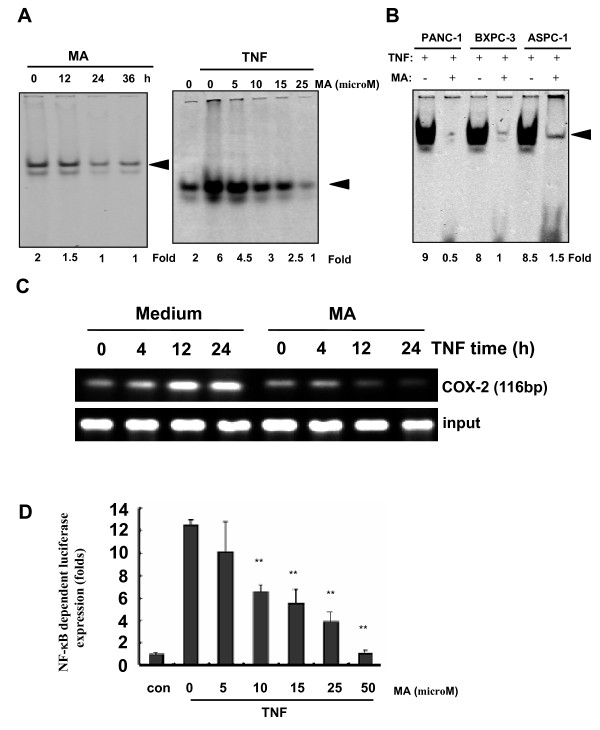
**MA inhibits TNFα-induced NF-κB activity in pancreatic cancer cells**. (**A**) MA suppressed endogenous NF-κB DNA binding activity (left) in a time-dependent manner and TNFα-induced NF-κB activity (right) in a concentration-dependent manner. Panc-28 cells were pretreated with indicated concentrations of MA for 6 h, incubated with or without 0.1 nM TNFα for 15 minutes, and then subjected to EMSA to examine NF-κB activation. Arrowhead indicates the DNA-binding activity of NF-κB. (**B**)MA suppressed TNFα-induced NF-κB activity in different pancreatic cancer cell lines. Pancreatic cancer cells were pretreated with or without 25 microM MA for 6 h, and then incubated with 0.1 nM TNFα for 15 minutes. The DNA binding activity of NF-κB was evaluated by EMSA. (**C**) MA inhibited the binding of NF-κB to the COX-2 promoter *in vivo*. Panc-28 cells were pretreated with 25 microM MA for 12 h and treated with 0.1 nM TNFα for the indicated time. The proteins were cross-linked with DNA by formaldehyde and then subjected to ChIP assay using an anti-p65 antibody with the COX-2 primer. (**D**) MA inhibited TNFα-induced NF-κB dependant reporter gene expression. 293 cells were transiently transfected with a NF-κB luciferase reporter gene. After transfection, cells were pretreated with the indicated concentrations of MA for 24 h, and then incubated with 0.1 nM TNFα for another 24 h. Cell supernatants were collected and assayed for luciferase activity as described in Materials and Methods. Results are expressed as fold activity over the activity of the vector control. *Column*, means of three experiments carried out with triplicate; *bar*, SD. **, *P *< 0.01, ***, *P *< 0.001 versus TNFα alone treated group.

NF-κB is a key regulator in cell apoptosis and TNFα-induced NF-κB activation is relatively well understood [24]. To determine whether MA induced cell apoptosis is mediated by NF-κB, we investigated NF-κB activation using different approaches in the presence of MA. Firstly, using EMSA assay, we demonstrated that MA alone could suppress endogenous NF-κB DNA binding activity in a time-dependent manner and also suppress the TNFα-induced NF-κB activation in a dose-dependent manner in Panc-28 cells (Fig. 3A). The strong inhibitory effects of MA on TNFα-induced NF-κB DNA binding activity were further confirmed in three more different type pancreatic cancer cell lines Panc-1, BXPC-3 and ASPC-1 (Fig. 3B). Secondly, we tested whether MA can suppress the *in vivo *DNA binding of TNFα-induced NF-κB to the COX-2 promoter using CHIP assay. We demonstrated that TNFα induced NF-κB binding to COX-2 promoter in a time-dependent manner and that MA markedly suppressed the binding of NF-κB to the promoter (Fig. 3C). Finally, similar results were also obtained by NF-κB-luciferase reporter gene assay. As shown in Figure 3D, MA significantly inhibited TNFα induced NF-κB reporter gene activity in a dose-dependent manner (Fig. 3D). Together, all of our results suggested that MA strongly inhibited TNFα-induced NF-κB activity in pancreatic cancer cells.

### MA inhibited TNFα-induced p65 nuclear translocation and p65 phosphorylation in pancreatic cancer cells

**Figure 4:**
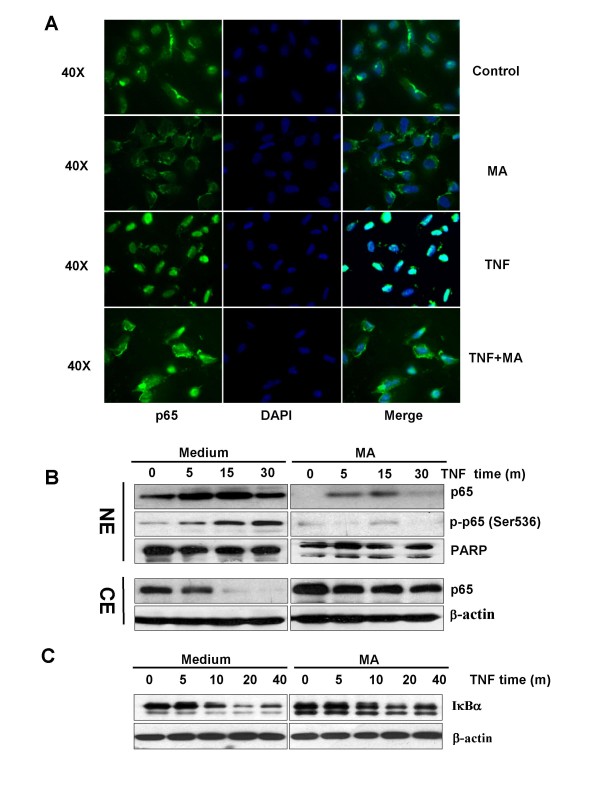
**MA blocks TNFα-induced p65 nuclear translocation, p65 phosphorylation and IκBα degradation in pancreatic cancer cells**. (**A**) MA inhibited TNFα-induced p65 nuclear translocation by immunofluorescence analysis. Panc-28 cells were pretreated with or without 25 microM MA, and then stimulated with or without 0.1 nM TNFα for 20 minutes. The localization of p65 was visualized by immunofluorescence analysis as described in materials and methods. Magnification, ×40. (**B**) MA inhibited TNFα-induced p65 nuclear translocation and p65 phosphorylation by Western blot. Panc-28 cells were incubated with 25 microM MA for 6 h and treated with 0.1 nM TNFα for the indicated time. Cytoplasmic extracts (CE) and nuclear extracts (NE) were analyzed by Western blot using the indicated antibodies. Anti-β-actin and anti-PARP antibodies were used as controls. (**C**) MA inhibited TNFα-induced IκBα degradation. Cells were pretreated with 25 microM MA, and then stimulated with 0.1 nM TNFα for 20 minutes. Whole cell extracts were prepared for Western blot analysis using indicated antibodies.

The effects of MA on p65 nuclear translocation and phosporylation were examined in pancreatic cancer cells. In the absence of TNFα, the majority of p65 was primarily located in the cytoplasm (Fig. 4A, Control). Upon TNFα treatment, almost all p65 signals were detected in the nucleus after 20 minutes of incubation (Fig. 4A, TNFα). MA inhibited TNFα-induced p65 nuclear translocation (Fig. 4A, TNFα+MA). To confirm the data generated by immunofluorescence staining, we performed Western blot analysis of p65 in nuclear extracts (NE) and cytoplasmic extracts (CE). As shown in Fig. 4B, total p65 and phosphor-p65 (p-p65^Ser536^) started to accumulate in a time-dependent manner when induced by TNFα, and the accumulation peaked in 15-30 minutes after stimulation of TNFα. On the other hand, total p65 in cytoplasmic extracts (CE) dramatically decreased after 15 minute treatment by TNFα (Fig. 4B, left). Treatment of MA inhibited TNFα induced p65 nuclear translocation and p65 phosphorylation (Fig. 4B, right), suggesting that MA suppresses TNFα induced NF-κB nuclear translocation and activation in pancreatic cancer cells.

### MA inhibits TNFα-induced IκBα degradation in pancreatic cancer cells

To understand whether treatment of MA affects TNFα-induced upstream of NF-κB/p65 pathway, we examined TNFα-dependent IκBα degradation. TNFα-induced IκBα degradation happens within 10 minutes of stimulation and reached a maximum at 20 minutes, but treatment of MA significantly inhibited TNFα induced IκBα degradation in pancreatic cancer cells (Fig. 4C).

### MA suppresses NF-κB-regulated gene expression in pancreatic cancer cells

**Figure 5:**
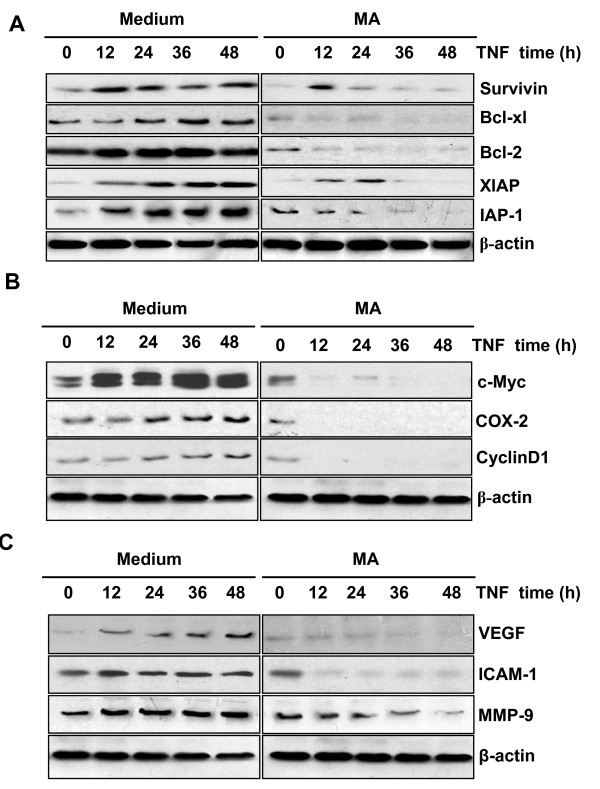
**MA suppresses TNFα-induced NF-κB downstream gene expression in pancreatic cancer cells**. Panc-28 Cells were pretreated with or without 25 microM MA for 6 h, then incubated with 0.1 nM TNFα for the indicated time, the whole cell extracts were prepared for Western blot analysis using antibodies for proteins involved in apoptosis (**A**), proliferation (**B**), and tumor metastasis (**C**).

NF-κB is known to regulate a variety of cell functions, including apoptosis, proliferation, invasion and angiogenesis through regulating target gene expression [24,25]. Using Western blot analysis, we examined the effects of MA on gene expression regulated by TNFα treatment and NF-κB activation. As shown in Fig. 5, MA inhibited TNFα-induced gene expression in a time-dependent manner. Specifically, MA suppressed the expression of anti-apoptosis genes, such as Survinin, Bcl-2, Bcl-xl, XIAP, IAP-1 (Fig. 5A), anti-proliferation genes, such as c-Myc, COX-2, Cyclin D1 (Fig. 5B), anti-invasion and angiogenesis related genes, such as ICAM-1, VEGF, MMP-9 (Fig. 5C).

### MA inhibits pancreatic tumor growth in a mouse xenograft tumor model by inducing apoptosis and suppressing NF-κB-mediated anti-apoptosis protein expression in vivo

**Figure 6:**
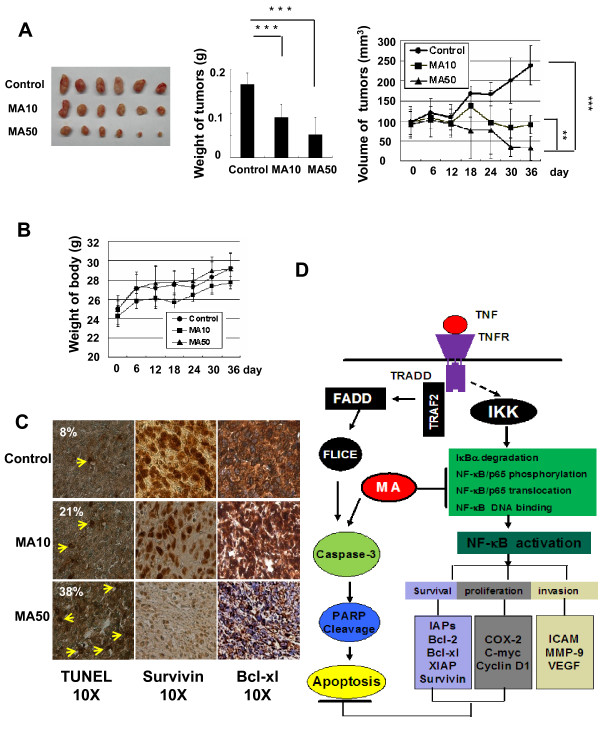
**MA suppresses pancreatic tumor growth, induces apoptosis, and inhibits the NF-κB-regulated anti-apoptotic gene expression *in vivo***. (**A**) MA inhibited pancreatic tumor growth in xenograft mouse model using Panc-28 pancreatic cancer cells in two different doses (10 mg/kg or 50 mg/kg). Both tumor volume and weight significantly decreased in MA-treated groups compared to DMSO control group in a dose-dependent manner. *Column*, mean; *bar*, SD (n = 6, **, *P *< 0.01, ***, *P *< 0.001 versus control). (**B**) MA had little effect on mouse body weight. No significant difference between MA-treated groups (10 mg/kg and 50 mg/kg) and the control group. (**C**) MA inhibited tumor apoptosis and suppressed the expression levels of NF-κB-regulated gene expression in xenograft mouse model. Solid tumor were fixed and embedded with paraffin. The 5 micro m sections were stained with specific TUNEL staining kit (left side), and antibodies for anti-apoptosis proteins, Survivin and Bcl-xl (arrowheads indicate the TUNEL signals, magnification, ×10). (**D**) A schematic diagram of mechanism by which MA increases pancreatic tumor susceptibility to TNFα. MA or TNFα alone can induce pancreatic cancer cell apoptosis through a caspase-dependant pathway (left). The apoptotic effect of TNFα is attenuated by MA due to NF-κB regulated anti-apoptosis gene expression (right). MA can suppress NF-κB activation therefore enhances TNFα induced apoptosis.

To examine whether MA can inhibit pancreatic cancer growth, potentiate cell apoptosis, and suppress NF-κB activity *in vivo*, we examined the effects of MA on the growth of xenograft implanted pancreatic tumors in athymic nu/nu mice. As shown in Fig. 6A, tumor growth was significantly suppressed by MA in a dose-dependent manner. The average tumor volume and tumor weight are significantly decreased in 10 mg/kg MA-treated group and in 50 mg/kg MA-treated group in a dose-dependent way, suggesting MA strongly inhibits tumor growth in xenograft mouse pancreatic tumor model. Moreover, both 10 mg/kg and 50 mg/kg MA-treated groups had little effect on body weight at the concentration tested (Fig. 6B), suggesting little toxicity of the compound at the tested concentration, which is consistent with previous studies [22,23].

To further uncover whether MA potentiates cancer cell apoptosis *in vivo*, we stained the solid tumor sections with apoptosis staining kit. As shown in Fig. 6C, application of MA in xenograft mouse model significantly increased apoptotic cells in a dose-dependent manner using TUNEL assay. The number of TUNEL-positive cells increased from 8 ± 3.2% positive nuclei in DMSO control group to 21 ± 5.41% and 38 ± 10.17% in 10 mg/kg and 50 mg/kg MA-treated groups, respectively. Furthermore, the expression levels of two NF-κB-regulated anti-apoptosis genes, Survivin and Bcl-xl were significantly decreased (Fig. 6C) by immunohistochemistry staining. The decrease of Survivin and Bcl-xl expression levels is dose-dependent, suggesting that MA can inhibit pancreatic tumor growth and induce tumor cell apoptosis through suppression NF-κB-mediated gene expression *in vivo*.

## Discussion

The aim of this study was to determine whether MA, a nontoxic microchemical ingredient and widely present in the diet, could sensitize pancreatic cancer to the treatment of TNFα. In various pancreatic cancer cell lines, MA or TNFα alone moderately inhibited cancer cell growth and induced apoptosis. When combined together, these anti-tumor cell effects were dramatically increased. To understand the underlying mechanisms, we demonstrate that MA could enhance TNFα induced caspase apoptotic pathway and suppress TNFα induced NF-κB activation (Fig. 6D). MA can inhibit TNFα induced IκBα degradation, block p65 nuclear translocation and phosphorylation, and finally, down-regulate the expression levels of NF-κB-mediated genes/proteins involved in proliferation (Cyclin D1, COX-2 and c-Myc), apoptosis (Survivin, Bcl-2, Bcl-xl, XIAP, IAP1), invasion (MMP-9 and ICAM-1), and angiogenesis (VEGF). In athymic nu/nu mice model, MA suppressed pancreatic tumor growth, induced apoptosis, and inhibited NF-κB-regulated antiapoptosis genes (Survivin and Bcl-xl).

Previous studies indicate that TNFα have multiple roles in pancreatic cancer [13,26,27]. Several evidences found that TNFα could induce pancreatic cancer cell apoptosis *in vitro *and *in vivo *[11,27]. However, it is also reported that pancreatic tumor secreting TNFα may be the crucial trigger of tumor recurrence and metastasis after surgical resection [8,28]. Many studies also showed that tumor secreted-TNFα could prevent pancreatic cancer cell from apoptosis mediated by exogenous TNFα by activating NF-κB signaling pathway [8,14]. Furthermore, the apoptosis potency of TNFα can be increased in certain tumors when NF-κB activity is inhibited [2,13,16,27,29-32]. Therefore, inhibition of NF-κB activation by pharmacologic approaches has become an attractive strategy for improving the anti-tumor activity of TNFα [2,10,13,27]. In the current study, we found that MA or TNFα alone showed moderate apoptosis activities in pancreatic cancer cell lines. However, MA dramatically increased cell apoptosis and inhibited NF-κB activity induced by TNFα in various pancreatic cancer cell lines, suggesting a synergistic effect of MA together with TNFα. And finally, we demonstrated that the expression levels of NF-κB-mediated downstream genes, including anti-apoptosis genes, proliferation genes, and metastasis related genes induced by TNFα, were all inhibited by MA in the pancreatic cancer cells.

In this study, we found that MA alone can effectively suppress pancreatic tumor growth, induce tumor apoptosis, and inhibit the expression levels of NF-κB-regulated anti-apoptosis genes in xenograft nude mouse models. Previous studies have shown that the secretion of TNFα was highly increased in pancreatic tumor tissues and even in serum *in vivo *[8,27,29]. Human plasma levels of TNFα were significantly higher in pancreatic adenocarcinoma patients (32.7 pg/ml) compared with chronic pancreatitis patients (3.2 pg/ml) and control group (<1.6 pg/ml; p < 0.01) [33], suggesting that MA could be an effective agent in the treatment of pancreatic cancer due to the high level of TNFα secretion in the patients.

## Conclusions

In summary, our data demonstrate that MA can potentiate the anti-tumor activity of TNFα, inhibit pancreatic cancer proliferation, invasion, and induce tumor cell apoptosis by increasing caspase-mediated apoptotic activation and suppressing NF-κB activity in pancreatic tumors (Fig. 6D). These results support the conclusion that MA together with TNFα could be a new promising nontoxic agent in the management of pancreatic cancer.

## Methods

### Materials

MA was synthesized in our laboratory. The antibodies used in this study were all obtained from Cell Signaling Technology (Danvers, MA). The terminal deoxynucleotidyl transferase (TdT)-mediated dUTP nick end labeling (TUNEL) was obtained from Millipore Biotechnology. Penicillin, streptomycin, MEM/F12, DMEM (high glucose) and fetal bovine serum (FBS) were obtained from Invitrogen. Bacteria-derived recombinant human TNFα, Tris, glycine, NaCl, SDS, and bovine serum albumin (BSA) were obtained from Sigma (St. Louis, MO).

### Cell lines and cell culture

The Panc-28 cell line was a generous gift from Dr. Bharat B. Aggarwal (The University of Texas M.D. Anderson Cancer Center Houston, TX). Panc-1, BxPC-3, AsPC-1 and A293 were obtained from American Type Culture Collection. Panc-28 cells were cultured in DME/F12 supplemented with 10% FBS. Other cells were cultured in DMEM supplemented with 10% FBS.

### Proliferation assay

The proliferation effect of MA and TNFα was determined by the MTS ((3-(4,5-dimethylthiazol-2-yl)-5-(3-carboxymethoxyphenyl)-2-(4-sulfophenyl)-2H-tetrazoliu m)) method following the manual of CellTiter 96 Aqueous One Solution Cell Proliferation assay (Promega) with VERSAmax microplate reader (Molecular Devices). Briefly, 5000 cells were incubated with MA for 6 h in triplicate on 96-well plates and then treated with 0.1 nM TNFα for 36 h at 37°C. Thereafter, 20 microL MTS solution-I was added to each well. After 2~4 h of incubation at 37°C, the OD was measured at 490 nm using a 96-well multiscanner.

### Transwell invasion assay

To test the effect of MA on cell invasion activity, we performed transwell invasion assay as previously described {Yi, 2008 #37}. Briefly, the starved cells (1 × 10^5^/well) were seeded onto the top chambers of the transwell (Corning, with an 8 micro meter pore polycarbonate filter insert) coated with 0.1% gelatin (Corning). The bottom chambers were filled with DMEM/F12 with 10% FBS supplemented with 0.1 nM TNFα. The top and bottom chamber medium contain the same concentration of MA. PANC28 cells were allowed to migrate for 12 h. Then scraped the cells on the top surface of membrane and stained, counted the cells on the bottom side of the membranes (migrated cells) using OLYMPUS inverted microscope.

### Live and Dead assay

To assess the cytotoxicity of MA and TNFα, we performed Live/Dead assay as previously described [16]. Briefly, 1 × 10^5 ^cells were incubated with 25 microM MA for 12 h, and then incubated with 0.1 nM TNFα for 24 h at 37°C. Cells were stained with Live/Dead reagent (5 microM ethidium homodimer and 5 microM calcein-AM) and then incubated at 37°C for 30 min. Cells were analyzed under a fluorescence microscope (DM 4000B, Leica).

### Annexin V and Tunnel assays

To detect whether MA induces pancreatic cancer cell apoptosis, we stained the treated cells with annexin V kit (sigma) as previously described [16]. Briefly, 1 × 10^6 ^cells were pretreated with 25 microM MA for 3 h, treated with 0.1 nM TNFα for 12 h, and then subjected to annexin V staining. The results were analyzed with a flow cytometer (FACSAria; BD Biosciences) Cell apoptosis was also measured by the TUNEL assay using an *in situ *cell death detection reagent (Millipore) as previously described. Stained cells were analyzed with a flow cytometer.

### Electrophoretic mobility shift assay

To assess NF-κB activation by TNFα, we performed electrophoretic mobility shift assay (EMSA) using the Odyssey Infrared EMSA Kit following the manufactiure protocol. Briefly, nuclear extracts were prepared as previously described [16]. Nuclear extracts (5 micro g/sample) were incubated with NF-κB IRDye™ 700 Infrared Dye Labeled Oligonucleotides, 5' AGTTGA**GGGGACTTTCCC**AGG C 3' and 3' TCAACT**CCCCTGAAAGGG**TCCG 5' (Boldface indicates NF-κB binding sites) in reaction buffers, for 30 min at 37°C. DNA-protein complex was separated from free oligonucleotide on 6.6% native polyacrylamide gels. The gels were visualized with Odyssey Infrared system and were quantitated using Imagequant software (LICOR Biosciences).

### NF-κB-dependent luciferase reporter gene assays

The effect of MA on TNFα-induced NF-κB-dependent luciferase reporter assays were performed as previously described [16].

### Chromatin immunoprecipitation (CHIP) assay

CHIP assay was performed as described previously with some modification. Panc-28 (2 × 10^7 ^cells) were incubated with 25 microM MA for 6 h and then treated with 0.1 nM TNFα for the indicated time. Cells were then cross-linked with formaldehyde, quenched with glycine, and sonicated on ice and centrifuged at 4°C. Mixtures were incubated with anti-p65 antibody with rotation at 4°C overnight and then incubated with 100 micro L of protein A beads at 4°C for 6 h. After gentle centrifugation (2000 rpm), beads were resuspended in 1 mL of wash buffer, washed 3 times. Finally, immunocomplexes were washed and eluted with elution buffer (500 μg/ml each) at 37°C for 30 minutes. Extracted and dissolved Immunoprecipitated DNA was prepared for PCR assays. PCR products were run on 1.5% polyacrylamide gel and stained with ethidium bromide. Stained bands were visualized under UV light and photographed. The primers corresponding to -434/-414 and -319/-337 of human COX-2 promoter sequence: 5' AAAGACATCTGGCGGAAACCT 3' and 5' AGGAAGCTGCCCCAATTTG 3', were used in the PCR reactions.

### Immunocytochemical analysis of NF-κB/p65 localization

The effect of MA on the nuclear translocation of p65 was examined by immunocytochemistry as previously described. Briefly, cells were pretreated with 25 microM MA following seeded on a gelatin-coated glass, after stimulation with or without 0.1 nM TNFα for 20 minutes, fixed with 4% paraformaldehyde after permeabilization with 0.2% Triton X-100. After being washed in PBS, blocked and then incubated with rabbit polyclonal anti-human p65 antibody at a 1/25 dilution overnight incubation at 4°C, washed, incubated with goat anti-rabbit IgG-Alexa Fluor 594 (Molecular Probes) at a 1/500 dilution for 1 h, and counterstained for nuclei with DAPI (50 ng/ml) for 5 min. Stained slides were mounted with mounting medium purchased from Sigma-Aldrich and analyzed under a fluorescence microscope.

### Xenograft mouse model of pancreatic cancer

Xenograft mouse models were performed as described. 4-5 week-old athymic nu/nu male mice purchased from SIBS, Shanghai. Maintain, use, and treatment of all animals in this study were in accordance with accepted standards of the Ethics Committee at ECNU. Mice weighing about 20 g were divided with six mice per group. Panc-28 tumor cells were s.c. injected (3.3 × 10^6 ^cells per mouse) into the mice. After the tumors had established (about 100 mm^3^), the mice were subcutaneous injected with 10 mg/kg or 50 mg/kg MA every two day. The control mice were injected with DMSO. The mice body weight and tumor sizes were recorded every two-day and the tumor size were determined by Vernier caliper measurements and calculated as length × width × height. After 36 days, mice with tumors were sacrificed.

### Histology and immnohistochemistry

Tumor were removed, weighed, fixed with 10% formalin, and embedded with paraffin. TUNEL assay was performed to detect apoptotic cells using the *in situ *Cell Death Detection kit from Chemicon according to the manufacture's instructions. The expression of Survin and Bcl-xl were examined using an immunohistochemical method described previously.

### Statistical analysis

Data are represented as mean ± SD for the absolute values as indicated in the vertical axis legend of figures. The statistical significance of differential findings between experiments and controls was determined by t-test or Dunnett test as implemented by SPSS10.0 software. P values smaller than 0.05 were considered statistically significant.

## Competing interests

The authors declare that they have no competing interests.

## Authors' contributions

CL, ZY, CZ, WQ and LW performed the experiments, WQ and JT synthesized the compound, CL, DL, ZY, LW, JT, MQ, JL, and ML analyzed the data, CL, JL and ML designed the experiments and prepared the manuscript. All authors read and approved the final manuscript.
